# Low back pain in military police activity: analysis of prevalence, associated factors, and ergonomics

**DOI:** 10.47626/1679-4435-2021-626

**Published:** 2021-12-30

**Authors:** Matheus Curcio Locatelli

**Affiliations:** Segurança Pública, Polícia Militar do Estado de Santa Catarina, Criciúma, SC, Brazil.

**Keywords:** low back pain, prevalence, ergonomics, police, dor lombar, prevalência, ergonomia, polícia

## Abstract

**Introduction:**

Low back pain is a frequent occupational complaint, corresponding to a considerable portion of leaves of absence that lead to economic loss. This symptom is frequently observed in military police officers, which carry around mandatory gear, which increases overload of the lumbar spine.

**Objectives:**

This study aimed to evaluate the prevalence of low back pain among military police officers, to identify associated factors, and to assess ergonomic hazards.

**Methods:**

This is a cross-sectional study, which analyzed the presence of low back pain, the degree of disability (with the Orwestry Disability Index), and possible associated factors in 2 subgroups of military police officers in the South region of the state of Santa Catarina.

**Results:**

Our sample consisted in 221 military police officers; 194 wore belt holsters and 27 wore drop leg holsters. The first group showed a higher prevalence of low back pain (74.2%) and pain chronification (70.1%). A higher prevalence of pain was observed in the extremes of age and also among police officers who had been on the job longer. The mean Orwestry Disability Index was higher in the group wearing belt holsters.

**Conclusions:**

Protection gear carried around by military police officers may be related to low back pain complaints; a better weight distribution of this equipment on the body may be beneficial in the prevention of low back pain. The drop leg holster was demonstrated to be a possible solution, allowing weight distribution to the lower limbs and decreasing overload of the lumbar spine due to equipment weight.

## Resumo

**Introdução:**

Lombalgias são queixas ocupacionais frequentes, correspondentes a uma parte considerável de afastamentos laborais que implicam, consequentemente, em prejuízo econômico. Esse sintoma é frequentemente observado nos policiais militares, que carregam consigo equipamentos obrigatórios atrelados ao corpo, os quais aumentam a sobrecarga sobre a coluna lombar.

**Objetivos:**

Este estudo objetivou avaliar a prevalência de dor lombar em policiais militares, identificar os fatores associados e avaliar os riscos ergonômicos.

**Métodos:**

Estudo transversal que avaliou a presença de dor lombar, o grau de incapacidade da dor através do índice de incapacidade de Oswestry e possíveis fatores associados em dois subgrupos de policiais militares do Sul do estado de Santa Catarina.

**Resultados:**

A amostra deste estudo consistiu em 221 policiais militares; 194 faziam uso do coldre de cintura, e 27, do coldre de perna. O primeiro grupo apresentou maior prevalência de dor lombar (74,2%) e cronificação da mesma (70,1%). Foi observada ainda maior prevalência de dor em extremas faixas etárias do estudo e também nos policiais que tinham maior tempo de serviço. A média de pontuação no índice de incapacidade de Oswestry foi maior no grupo que usava coldre de cintura.

**Conclusões:**

Os equipamentos de proteção atrelados ao corpo do policial militar podem estar relacionados às queixas de dor lombar; uma melhor distribuição do peso desses equipamentos no corpo pode trazer benefícios na prevenção das dores lombares. O coldre de perna demonstrou ser uma possível solução, permitindo uma distribuição de peso nos membros inferiores e diminuindo a sobrecarga dos equipamentos sobre a coluna lombar.

## INTRODUCTION

It is estimated that approximately 60% to 90% of the population will have an episode of low back pain in their life,^1^ and this is the second most common complaint in clinical practice after headaches.^2^ Studies demonstrate that 37% of cases of low back pain are related with occupational factors.^3^

Occupational low back pain corresponds for the main cause of disability in workers aged 45 years or less; this population performs work activities, which reflects an important problem that is not only social but also economic.^4^ It is also the main cause of work-related health disorders and absenteeism.^2^

Low back pain may be classified according to its duration: pain lasting less than 6 weeks is considered acute; between 6 and 12 weeks, it is considered subacute; and pain that lasts more than 12 weeks is considered chronic.^5^

Many conditions have been associated with low back pain, contributing to its chronification (some of them still lack full scientific support), such as psychosocial factors, job dissatisfaction, obesity, smoking habits, schooling level, physically demanding jobs, sedentary behavior, depressive syndromes, labor disputes, genetic and anthropological factors, postural habits, and climate changes.^6^ Studies also indicate that driving vehicles for more than 4 hours per day is a possible risk factor for low back pain.^7^

The peculiar activity of military police officers exposes them to a series of the aforementioned factors, which puts them at risk for developing low back pain. Although there are administrative activities within the military police forces, most of these professionals have an operational activity and carry an arsenal of equipment — ballistic vest, duty belt, firearm — during the whole working day, in addition to having long work shifts of up to 12 hours.^8,9^

Notably, ergonomics thus represents a component with crucial importance in the military police activity. The literature describes the instrumentality of work as comprising tools, equipment, and devices used by the worker to achieve wellbeing on the job.^10^ Tavares Neto et al.^9^ described, on a study considering data on low back pain in military police forces, that this instrumentality of work corroborates a favorable scenario for the appearance of this symptom.

A 2010 study performed in the state of Bahia with military police officers reports low back pain as the main reason for leaves of absence among these professionals, affecting 50% of military officers aged 35 years or more.^9^

This study aimed to assess the prevalence of low back pain in military police officers and identify possible related factors. On an ergonomic analysis, gear used by different police subdivisions was compared in order to verify possible interactions between equipment and low back pain. The disability imposed by low back pain was evaluated with the Oswestry Disability Index (ODI).

The main goals of this study were to raise hypotheses that may contribute to prevention strategies against low back pain in these professionals, thus dampening this important cause of leaves of absence and consequently decreasing the number of abstentions for this reason, reducing the economic burden to the State.

## METHODS

This is a cross-sectional study performed with military police officers from the South region of the state of Santa Catarina between August and October 2016.

This study included all military police officers on active duty (those who had not yet completed the required years of service for reserve retirement), aged 18 years or older, who were on operational duty doing vehicle patrol shifts, and who accepted to participate in the study by signing a consent form. Military on administrative duty, doing motorcycle patrolling, or in other subgroups such as the K-9 and cavalry units were excluded from the study. Police officers with chronic pathologies were excluded from the study; female officers were also excluded due to hormonal and pregnancy-related issues that could influence biomechanics of the spine, which has been well-documented in the literature.^11^ The goal of these inclusion and exclusion criteria was to provide a sample as homogeneous as possible, since the Santa Catarina State Military Police (PMSC) has various groups of officers performing different activities. These filters allowed the formation of 2 similar groups as to their end activity, with the only basic difference being their firearm gear — one group wore belt holsters and the other wore drop leg holsters. While the first piece of equipment is wrapped around the worker’s pelvic girdle, the second is strapped to the middle third of one or both thighs.

It is worth noting that these 2 groups of military police officers had comparable working hours, usually working in 12-hour shifts with 36 hours off.

Data were collected through an interview with the researcher, with a form for assessing possible risk factors associated with low back pain, such as time of service, age, and obesity. The ODI was also used for evaluating the functional capacity of military officers regarding low back pain.

The ODI has percentage scores of 1 to 100, which are stratified considering disability due to low back pain as follows: 0-20% — minimal disability; 21-40% — moderate disability; 41-60% — severe disability; 61-80% — crippled; and 81-100% — bed-bound.^12^

All types of pain, irradiated or not, between the inferior costal margin and above the gluteal line were accepted as low back pain for inclusion in this study, at any moment of employment by the military police. General characteristics of the pain, such as duration and irradiation, were also considered.

Epi Data 3.1 software was used for statistical analysis of the data. Statistical significance considered p < 0.05, and a 95% confidence interval (95%CI) was used.

This project was developed after authorization by the General Commander of the PMSC and approval by the Ethics Committee of Universidade Federal do Rio Grande do Sul (opinion No. 2.995.477).

## RESULTS

This study evaluated 221 military officers on exterior operational duty who performed vehicle patrolling — 28.8% of all military officers in the Extreme South of the state of Santa Catarina. The Extreme South region had, at the time of this study, 767 military officers.

Two military police officers with hypothyroidism were excluded from the study, in addition to an officer with hip dysplasia, as these are chronic diseases that may constitute confounding factors for low back pain. None of the police officers refused to participate in the study.

Out of the evaluated sample, 194 (87.8%) military officers wore belt holsters and 27 (12.2%) wore drop leg holsters. Low back pain was reported by 150 (67.9%) military officers; 144 of them wore belt holsters and 6 wore drop leg holsters. The prevalence of at least one pain episode was 74.2% (144) among officers wearing belt holsters and 22.2% (6) for those wearing drop leg holsters (p < 0.0001). A total of 71 interviewees reported not having low back pain complaints since they became military police officers.

Regarding chronic pain, 106 (70.6%) of the interviewees stated that their pain lasted for more than 12 weeks (characterizing chronic pain), whereas 44 said their pain lasted for less than 12 weeks. Considering officers who reported chronic pain, 101 (70.1%) wore belt holsters, thus suggesting a higher prevalence of chronic pain among military police officers who used this equipment. However, this finding was not considered statistically significant (p = 0.671) (Table 1).

**Table 1:** Relationship between low back pain and holster use

Low back pain > 12 weeks	Yes n (%)	No n (%)	Total n (%)
Belt holster	101 (70.1)	43 (29.9)	144 (100.0)
Drop leg holster	5 (83.3)	1 (16.7)	6 (100.0)

The mean age of the studied population was 34 years, with a minimum age of 22 and maximum age of 52 years (standard deviation [SD] ± 6.6). The mean time of service considering the sample was 11 years (median value, 9 years), where the minimum time of service was 3 years and the maximum was 31 (SD ± 7.7) Mean body mass index (BMI) was 26.82, with the minimum value being 19.59 and the maximum value, 40.08.

A total of 10 police officers reported having pain before joining the military. Eight of these officers wore, at the moment of the study, the belt holster; 2 wore drop leg holsters. Seven of the officers wearing the belt holster answered that police activity worsened their pain intensity, while no officer wearing the drop leg holster reported worsening of their pain after joining the military (p = 0.035).

Considering the ODI, the mean score was 10 and the median was 8, while the minimum score was 0 and the maximum score was 42 (SD ± 8.6).

The maximum ODI score for officers wearing the belt holster was 42 points, whereas that for the drop leg holster was 36. Both holsters had a minimum score of 0. Military police officers who wore belt holsters had higher mean ODI scores, indicating higher disability in this group in relation to the drop leg holster (p = 0.001). The mean ODI score of officers wearing the belt holster was 10.7 (SD ± 8.4); for the drop leg holster, it was 4.81 (SD ± 7.8). The level of moderate disability with the belt holster was 13.4%, whereas that for the drop leg holster was 3.7% (p = 0.227) (Table 2).

**Table 2:** Relationship between disability due to low back pain according to the Oswestry Disability Index (ODI) and holster types

ODI score	0-20 n (%)	21-40 n (%)	42-60 n (%)	Total n (%)
Belt holster	167 (86.1)	26 (13.4)	1 (0.5)	194 (100.0)
Drop leg holster	26 (96.3)	1 (3.7)	0 (0.0)	27 (100.0)

A relationship of age group, obesity, and time of service with low back pain was also observed, showing some associations within groups.

The age variable, for example, was divided into 3 groups; the prevalence of low back pain was assessed in each one of them and it was higher in the older age group (p = 0.076) (Table 3).

**Table 3:** Relationship between age group and low back pain

Age group	20-30 years n (%)	31-40 years n (%)	41-52 years n (%)	Total n (%)
With low back pain	47 (65.3)	62 (63.3)	41 (80.4)	150 (67.9)
Without low back pain	25 (34.7)	36 (36.7)	10 (19.6)	71 (32.1)

The extreme age group presented a higher mean ODI score, in addition to being the only group that reached the maximum score of 42 (p < 0.0001). The mean ODI score for this age group was 14.04, while that for the youngest age group was 9.44 and that for the intermediate age group was 8.37.

The prevalence of low back pain was also evaluated regarding time of service. Time of service was also divided into 3 groups: the first included military officers with 1 to 10 years of service (the minimum time of service was 3 years); the second group included those with 10 to 20 years of service; the last one comprised officers with 21 to 31 years of service at the PMSC. Pain prevalence was higher among those with longer active military police service (p = 0.060). Out of all officers, 78.4% of those who belonged to the group with 21-30 years of service reported low back pain; this percentage was 73.3% for officers with 11-20 years or service and 61.6% for those with 1-10 years of service.

The longer the time of service, the higher the ODI score. Military officers with 21 or more years of active service had a mean ODI of 13.96. Military officers with less time of service had a mean score of 8.35; the mean score for the intermediate group was 10.22 (p < 0.0001).

Military officers who were overweight or obese to some degree (according to their BMI) also presented a higher frequency of pain (p = 0.001). Out of the officers who reported low back pain, 109 (72.7%) were overweight and 17 (11.3%) were obese to some degree. Out of the officers who reported no low back pain, 39 (54.9%) were overweight and only 5 (7%) were obese to some degree.

## DISCUSSION

**Figure 1:**
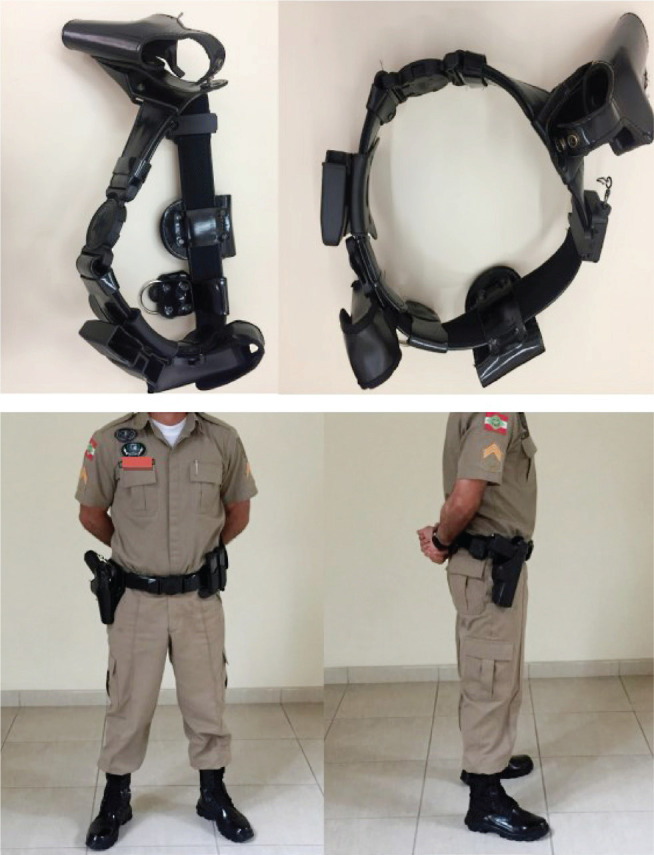
Belt holster.

**Figure 2:**
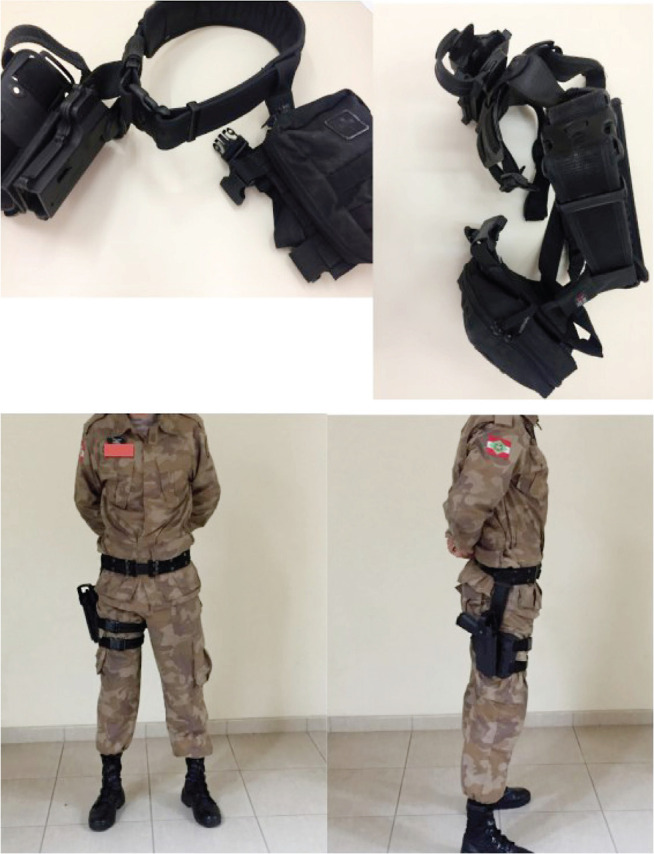
Drop leg holster.

PMSC had, at the time of this study, the belt holster as standard equipment for carrying firearms during patrol (Figure 1); the drop leg holster (Figure 2) was dedicated to specialized forces such as motorcycle patrol, cavalry and K-9 units, and the tactical group; the latter — due to their similar activity to standard vehicle patrol — was selected for comparison with officers wearing the belt holster. In 2016, proportionately more officers were allowed to wear only the belt holster because standard vehicle patrol largely outnumbers other types of police service described in this study.

PMSC officers have a 40-hour work week divided into shifts, depending on the type of police work. Standard vehicle patrol is performed in daily 8-hour shifts, 5 days a week, or continuous 12-hour shifts separated by 24 or 48 off hours. This last shift roster is also observed in the tactical group.

Both groups carry, as their standard gear, a ballistic vest weighting approximately 2.50 kg (2.25 kg for the smaller size, 2.60 kg for the medium size, and 2.95 kg for the larger vest), and a holster with suspenders. Since the ballistic vest is worn by all police officers, the difference is mainly observed when considering the gear used for carrying other objects — the holster. The belt holster, which weights 1,00 kg, holds a 0.90-kg firearm, loaded magazines (usually 2, in addition to that already in the firearm) — each weighting 0.35 kg —, 0.30-kg handcuffs, and a 0.10-kg pepper spray bottle; in total, approximately 6.00 kg of gear are attached to the officer’s body. The drop leg holster weights 1.00 kg and can be used on one leg or distributed as 0.50 kg on each leg. This holster allows better distribution of equipment between lower limbs on the mid-thigh. Both holsters are made of synthetic material — a polymer —, and the belt holster is lined with leather.

Few studies are available in the Brazilian literature regarding military police activity or the use of police gear. A similar work was performed with the Canadian Police in 1998 by Brown et al.,^13^ comparing 4 populational groups. One of the groups comprised police officers who wore the belt holster while doing vehicle patrol, and the others consisted in mounted police officers, administrative officers, and the general population. Although previous studies demonstrated that pain prevalence among police officers was higher than that in the general population, this fact was not observed in this 1998 study; the prevalence of pain among Canadian police officers wearing belt holsters was 52.4%,^13^ compared to 67.9% in our study. The Canadian study does not mention characteristics of the duty belt, such as weight, comfort, and adjustability, or the weight of the firearm.

In 2014, dos Santos et al.^14^ performed a study in the state of Paraná with 43 military police officers ranging operational and administrative services. Although it had a small number of participants, the study found statistical significance for a low back pain prevalence of 45% in the operational group. The study did not mention the characteristics of the duty belt worn by police officers. The prevalence of pain was significantly smaller than that found in the results of this study. This divergence may be partially explained by the mean age of military police officers in both studies. While military police officers in this study had a mean age of 34.26 years, those in the study by dos Santos et al.^14^ had a mean age of 28.6 years. The BMI reported by Santos et al.^14^ was 25.1, while in this study it was 26.82. Although BMI is not the best measure for assessing body composition, as it does not consider important differences in weight such as body mass and fat — which has considerable importance in the pathogenesis of musculoskeletal pain^15^ —, it is still routinely used by scientific studies. The small difference in BMI observed between groups possibly had little influence on the difference in low back pain prevalence in both studies; the mean BMI values in the study by dos Santos et al.^14^ and in this study were in the overweight range.

Still in the Canadian study, 8.5% of the sample reported having low back pain before joining the force.^13^ Our percentage was lower for this answer — 4.5%.

In a study performed in 1993 in the United Kingdom with 80 police officers who spent more than half their working days driving police cars, Gyi & Porter^16^ found a considerable statistical association between driving motor vehicles during most of the workday and low back pain. Driving motor vehicles for long periods with a fixed posture and the vibration of the vehicle are factors that may be involved with low back pain. Obviously, many other factors may also be associated with these elements in low back pain complaints, such as excessive weight, gender, lifestyle habits, and height.^16^ In the peculiar activity of military police officers, in addition to the ballistic vest and the holster and associated equipment (which significantly increase the load on the spine, especially on the lumbar region), it is also important to mention the physical and mental stress that is inherent to the profession.

The prevalence of pain in this study was considerably higher among military police officers who wore belt holsters when compared to those wearing drop leg holsters: 74.2 and 22.2%, respectively. Only 5 officers wearing drop leg holsters reported pain lasting for more than 12 weeks, thus characterizing chronic pain; this number was 101 among those who wore belt holsters. Pain disability in this study was assessed through the ODI. Whereas only one (3.7%) officer who wore the drop leg holster was classified as having moderate disability, 26 (13.4%) of those wearing drop leg holsters had moderate disability. Moreover, no military police officers wearing drop leg holsters had severe disability, while 1 officer who wore a belt holster was classified as such.

Problems with the use of holsters and police activity are not exclusive to our military police forces, being described by studies in Canada and the United States of America (USA) mentioned by the American Occupational Safety and Health Administration (OSHA). OSHA indicates that low back pain is a common problem among police officers and that complaints regarding the duty belt are frequent, which represents a problem that affects health and safety. It also mentions that the holster shank and edges, in addition to other pieces of attached equipment, put pressure over the area in contact with the skin, resulting in low back pain. In the USA, a police officer carries various pieces of equipment attached to the holster, such as the weapon, handcuffs, a flashlight, a baton, the radio, and pepper spray; the complete gear may weight 9.00 kg. The same report indicates the discomfort caused on the lumbar spine by a holster equipped with all these items, especially when the officer is seated in a police vehicle; another issue would be the fact that this discomfort caused by the belt holster when sitting in the vehicle tends to push the officer forward in his seat, which makes him adopt a non-ergonomic position.^17^ In the long term, this vicious posture will lead to lumbar muscle fatigue, with consequent tension and hypoxia contributing to pain chronicity.^18^

Hoy et al.,^19^ in a 2019 epidemiological study, mentioned that the prevalence of low back pain increases with age, reaching its peak at around 60 to 65 years.^19^ Dionne et al.^20^ also declared that the prevalence of more severe types of pain increased with age. This study corroborates these data. Here, the prevalence of pain increased with age, reaching 80.4% of military officers in the extreme age group (p < 0.0001). Disability according to the ODI was also higher in this group, with a mean score of 14.04 points, while the younger age group had a mean score of 8.37 (p < 0.0001).

No studies available in the literature describe the association between years in the military police force and low back pain. Studies considering diverse groups of workers show divergences regarding years of service and low back pain perception. An occupational study with railroad workers showed that pain prevalence was significantly higher in workers with more than 10 years of service,^21^ while others indicate no association^22^; others, still, report a decrease in pain with years of service due to a growing experience in performing one’s activity in a way that provides more comfort and adjustment to the activity.^23^ A strong statistical association was found in this study between years of service and low back pain. The prevalence of low back pain in military officers with longer time of service (21-31 years) was 78.4%; among military officers with shorter time of service (1-10 years), it was 61.6% (p < 0.0001). ODI scores of the evaluated military officers were also proportionately higher with the increase in years of service. The mean scores for 1-10, 11-20, and 21-31 years of service were 8.35, 10.22, and 13.96, respectively (p < 0.0001).

The studies identified in this work did not advocate for the drop leg holster in comparison with the belt holster. However, considering some references that associate the belt holster with a higher prevalence of low back pain, alternatives were proposed considering a smaller overload of the police officer’s lumbar spine. For example, transferring equipment from the duty belt to the ballistic vest, such as the radio (which is already done in the PMSC) and handcuffs in order to reduce the weight carried around the waist. Kathy Espinoza points out, in a brief article discussing ergonomic issues related to the duty belts of American police officers, that a better weight distribution reduced pain experiences in 87% and pain intensity in 62% in case of complaints; it also made specific activities easier, such as climbing stairs.^17^ This study showed that military police officers who wore drop leg holsters reported less low back pain through the years of active duty than those who wore belt holsters; as mentioned by other studies, it is possible that a better distribution of the gear on the body reduces overload of the spine, thus generating les tension and consequently less risk of musculoskeletal pain.

This author does not advocate for the extinction of the belt holster nor the standardization of the drop leg holster in the PMSC. However, measures such as padding (on the top and bottom of the belt holster), adjustability, the use of synthetic materials that are better adapted to the waist, and a better distribution of the attached equipment (which is currently mostly attached to the belt/thigh holster) could be taken for achieving better weight distribution, reducing overload of specific areas such as the lumbar spine — these contributions would very probably have a positive impact in preventing low back pain in military police activity.^17^ An ergonomically viable and well-adjusted drop leg holster would help in this weight distribution, reducing overload of the lumbar spine.

Another aspect to be questioned consists in the shift rosters, since long working hours with no breaks for removing gear are mentioned as one of the factors that may aggravate low back pain. Walsh et al.^24^ have reported a positive relationship between driving for more than 4 hours per day and low back pain. Therefore, the possibility of 2 breaks with stretching exercises duly taught to the troops during service would also contribute to preventing low back pain.^24^ The promotion of sports activities and strategies for strengthening the dorsal muscles are also well-known aspects of low back pain prevention.^25^ Finally, patrol vehicle maintenance and regular adjustments of the vehicle seats considering their wear and tear could provide better postures to the officers driving them, avoiding forward dislocation of the body during working hours, which is also considered harmful by the reviewed literature.^26^

A limitation of this study is the small number of military police officers authorized to use the drop leg holster at the time of data collection.

## CONCLUSIONS

Military police activity has been reported, by some studies, as being associated with low back pain. This study observed a prevalence of low back pain, at any given moment of the career as military police officer, of 67.9% of the studied population. Some associations were also noticed, such as increases in the prevalence of these symptoms with age, BMI, and time of service. The ODI (disability index used in this study) also showed higher scores for the population with more years of service, as well as those wearing belt holsters.

Few studies are available, especially considering the Brazilian literature, on diseases and equipment regarding state military police forces. Studies available at the moment (most of them being international) revealed low back pain problems, suggesting a possible association of this type of pain with the mandatory gear used by these professionals. Efforts have been employed for police officers to reduce the load on the lumbar spine without hampering the use of protective gear that provides safety and a quick approach when needed.

The belt holster, in this study, showed a higher prevalence of pain and disability when compared to the drop leg holster. Other studies demonstrated that a better distribution of weight contributed to a reduction in low back pain. Therefore, the drop leg holster may help reduce the overload of the police officer’s lumbar spine, reducing the prevalence of low back pain. Studies with higher statistical relevance may aid in confirming this hypothesis.
